# Scalable processing and capacity of Si microwire array anodes for Li ion batteries

**DOI:** 10.1186/1556-276X-9-417

**Published:** 2014-08-21

**Authors:** Enrique Quiroga-González, Jürgen Carstensen, Helmut Föll

**Affiliations:** 1 Institute for Physics, Meritorious Autonomous University of Puebla (BUAP), Puebla 72570, Mexico; 2 Institute for Materials Science, Christian-Albrechts-University of Kiel, Kiel 24143, Germany

**Keywords:** Silicon microwire, Battery anode, High storage capacity, Scalability, Lithium ion battery, Micromachining

## Abstract

Si microwire array anodes have been prepared by an economical, microelectronics compatible method based on macropore etching. In the present report, evidence of the scalability of the process and the areal capacity of the anodes is presented. The anodes exhibit record areal capacities for Si-based anodes. The gravimetric capacity of longer anodes is comparable to the one of shorter anodes at moderate lithiation/delithiation rates. The diffusion limitation of the lithium ions through the electrolyte in depth among the wires is the limiting factor for cycling longer wires at high rates.

**PACS:**

82.47.Aa; 82.45.Vp; 81.16.-c

## Background

Silicon as anode material for Li ion batteries has a theoretical capacity of 4,200 mAh/g, more than ten times the capacity of standard graphite anodes. Microstructured Si in wire-shape overcomes problems caused by its four-fold volume expansion during its lithiation, allowing capacity stability over hundreds of cycles [1,2]. Arrays of Si wires have been intensively studied in the latest years as alternative anodes for Li ion batteries. Those arrays have been prepared by three major techniques:

(1) Vapor-liquid-solid (VLS) technique, using mostly ‘Au droplets’ as catalytic growth sites [1,3,4].

(2) Metal-assisted chemical etching of single-crystalline silicon [5,6].

(3) Electrochemical macropore etching followed by chemical over-etching (the technique presented in this work, discussed in detail later) [7-9]. The wires produced in this way are 3 to 20 times thicker than most of the reported nanowires, which have diameters in the 50- to 300-nm range.

With the first technique, nanowires usually in a random arrangement are obtained. This production process is limited with respect to the wire density, diameter control, wire length, and array stability. Moreover, an efficient low-resistivity connection to a current collector is not easy with this technique. Method 2 overcomes some problems of technique 1, and may be easier than method 3 from a process point of view, but has a number of limits with respect to optimizing the array geometry and attaching to a current collector. For the moment, there are no reports of pores or wires with modulated diameter by method 2, and thus, for the moment, it is not possible to fabricate interconnected wires forming a free-standing array of long wires. Having a free-standing array is important for the deposition of a mechanically stable metal contact at one side.

A new concept of Si anodes has been developed by technique 3, which consists of an array of Si microwires embedded at one end in a Cu current collector [9]. The capacity of the anodes is very stable over 100 cycles [2] and breaks all the records when considering the capacity per area (areal capacity) [10]. In the present work, the scalability of the production process will be discussed. As will become clear in the following lines, the capacity of the anodes is also scalable, with certain limits in the cycling rate.

## Methods

**Figure 1:**
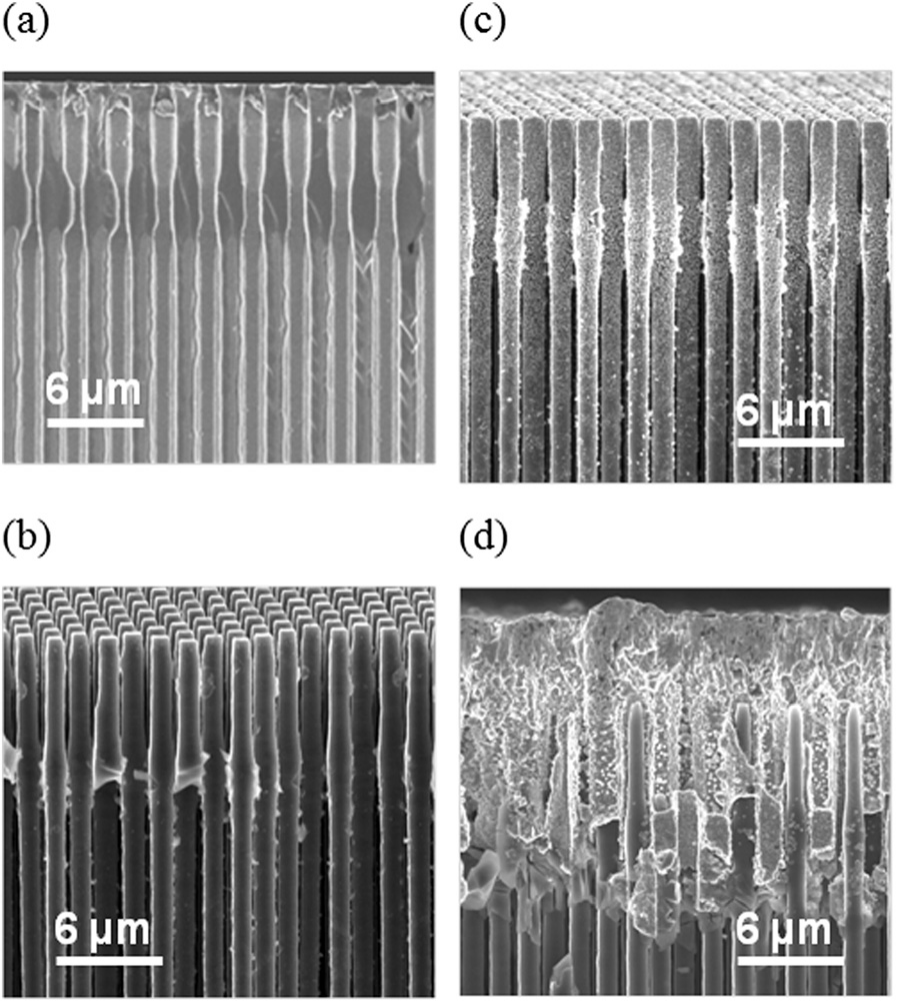
**Process steps for the production of Si microwire anodes. (a)** Electrochemical etching of macropores with modulated diameters. **(b)** Chemical over-etching of the pores to produce wires. **(c)** Electroless deposition of a Cu seed layer. **(d)** Electrochemical deposition of the Cu current collector.

The production process of the Si microwire anodes, depicted in Figure 1, consists of four main steps: (a) electro-chemical etching of macropores with modulated diameters. Sections with narrower diameters are created in order to produce (two) stabilization planes in the final wires. The starting material is Si wafers with a structure of pits defined by contact lithography. (b) The second step is chemical over-etching in KOH-based solutions of the pore walls; this step is done until the pores merge and wires remain. Commonly, the wires are produced with a diameter of around 1 μm. (c) The third step is electroless deposition of a Cu seed layer until certain depth. (d) The fourth step is electrochemical deposition of Cu on the Cu seed layer to create a current collector of the final anode. After this step, the anode is separated from the Si substrate by pulling from the Cu layer. Additional information of the fabrication process can be found in [9].

**Figure 2:**
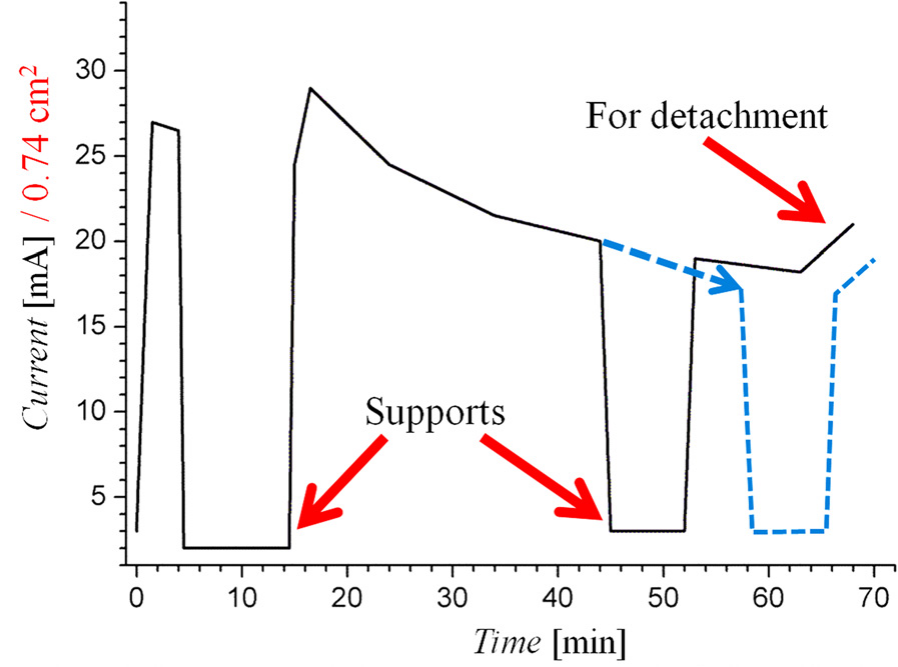
**Current profile used for the electrochemical etching of pores to produce wires.** The solid line indicates the profile used for the fabrication of the ‘standard’ wires of 70 μm in length. The dashed line indicates the case for producing longer wires.

A standard recipe for the fabrication of the anodes is as follows: (a) The electrochemical etching process of pores for the production of wires is done galvanostatically using the current profile shown in Figure 2 (solid line). p-type Si wafers with resistivity of 15 to 25 Ω cm are used, which are previously pre-structured with a quadratic array of pits with 3-μm pitch by contact lithography, reactive ion etching, and chemical anisotropic etching. The electrolyte consists of 5 wt% hydrofluoric acid (HF) in N,N′-dimethylformamide (DMF) and 8.2 g polyethyleneglycol (PEG) 3400 per liter electrolyte. The electrolyte temperature is kept constant at 17°C, while it is pumped through the etching cell at a rate of 600 mL/min. (b) After their production, the pores are over-etched to produce the desired wires. A common etchant is composed of 100 mL of a 0.45 wt% aqueous solution of KOH and 2 g of PEG 3400. The temperature is kept at 50°C. (c) The solution for the chemical deposition of Cu is prepared with 2 mL HF 48%, 98 mL H_2_O, and 1.9 g CuSO_4_ · 5H_2_O. The deposition is performed at 30°C. (d) The electrochemical Cu deposition is performed using a solution composed of 2.5 g CuSO_4_, 9.6 mL H_2_SO_4_, and 100 mL H_2_O. The deposition is done with a constant current of 5 mA/cm^2^ at 20°C. Standard anodes have Si microwires with quadratic cross section of 1 μm × 1 μm and length of 70 μm [2].

Battery cycling tests were performed using half-cells, with Li metal as counting and reference electrode. The separator was a glass fiber filter from Whatman (Piscataway, NJ, USA), with pores of 1 μm. The electrolyte was LP-30, consisting of dimethyl carbonate and ethylene carbonate (1:1) plus 1 mol/L of LiPF_6_. The tests were done with a BatSMALL battery charging system from Astrol Electronic AG (Othmarsingen, Switzerland). The anodes were cycled in a galvanostatic/potentiostatic mode, for which the voltage limits 0.11 V for lithiation and 0.7 V for delithiation were set. By this mode, when the voltage limit is reached, the cycling is switched to potentiostatic mode, and this mode finishes when the current has decreased to 10% of its initial value or when the capacity limit is reached.

SEM observations were performed with an Ultra Plus SEM from Zeiss (Oberkochen, Germany).

## Results and discussion

### Scalable processing

**Figure 3:**
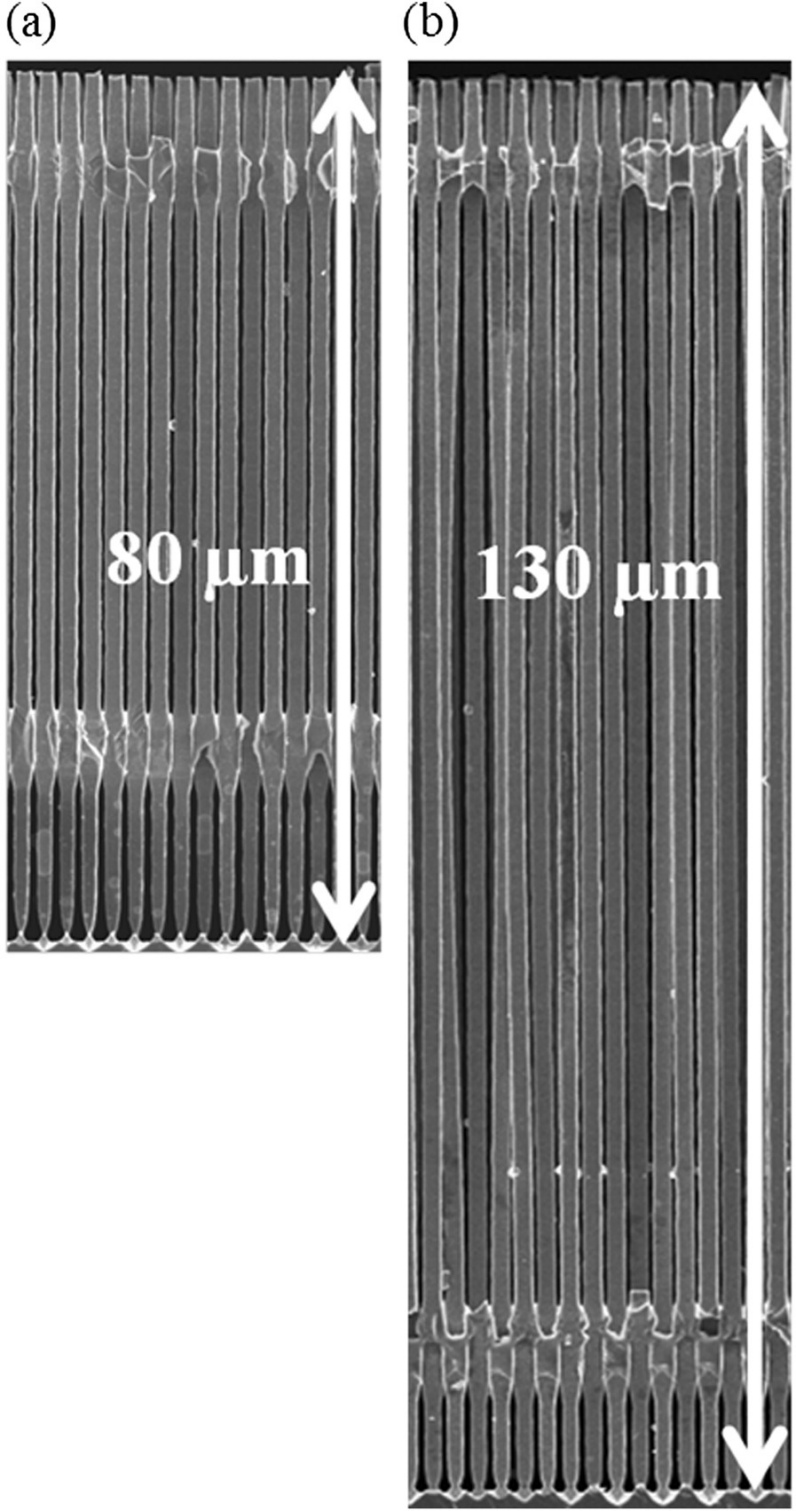
Si microwires produced with different lengths: (a) 80 μm and (b) 130 μm.

**Figure 4:**
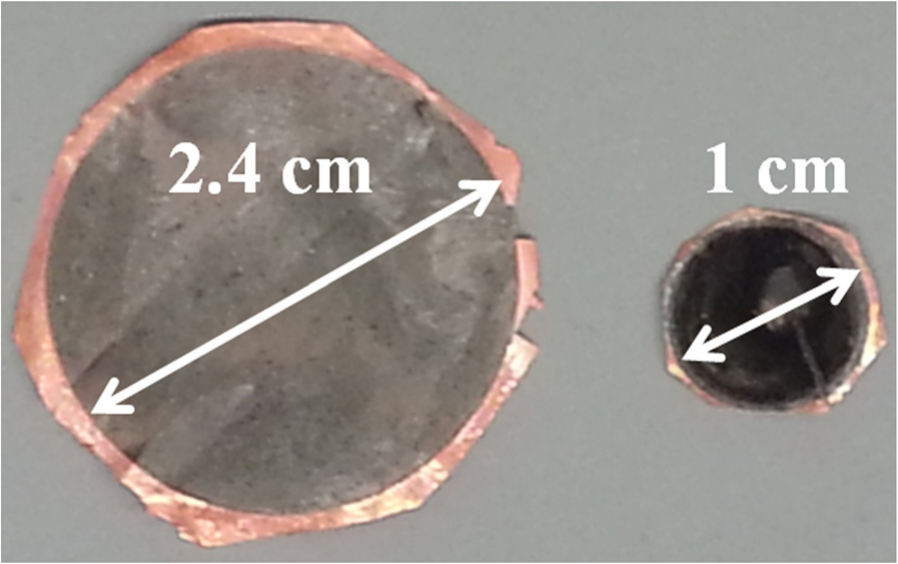
**Si microwire anodes produced in different areas.** Anodes with diameters of 2.4 and 1 cm are shown.

Aiming to prove that the previously described method is scalable to produce anodes with longer microwires or larger areas, different anodes were prepared. To prepare anodes with different wire lengths, the main parameter to be varied is the electro-chemical etching time between the two narrow sections of the pores. The current profile of Figure 2, in dashed line, is used to prepare larger wires than the standard ones; for this purpose, the etching time has been extended. It is clear that additionally the current density has to be reduced in depth in order to take into account the diffusion limitation of etchant. In depth, as there is less etchant available, less electric current has to be applied to avoid excessive parasitic currents and undesired effects like ‘lift-off’ of the porous layer. As examples, Si microwire arrays of lengths of 80 and 130 μm are shown in Figure 3 a and b, respectively.To produce anodes of different areas, also the main parameter to be varied is the etching current. The necessary etching current can be known by multiplying the current density (described in Figure 2) by a constant factor scaled according to the desired size of the anode. The scalability of the area may sound trivial, but it requires intense engineering work. Special care has to be taken about the temperature of the etching system when etching for large anodes, since a big portion of the consumed power is transformed into heat. The electrochemical etching process is temperature sensitive. Two examples of anodes of different sizes are shown in Figure 4. In principle, anodes as big as the size of the precursor Si wafers can be obtained. The rest of the steps for the production of anodes remains unaltered for longer/shorter anodes or for up/down scaling. Just the current for the electrochemical deposition of Cu has also to be scaled up/down in direct proportion to the size of the anodes.

### Scalable capacity

**Figure 5:**
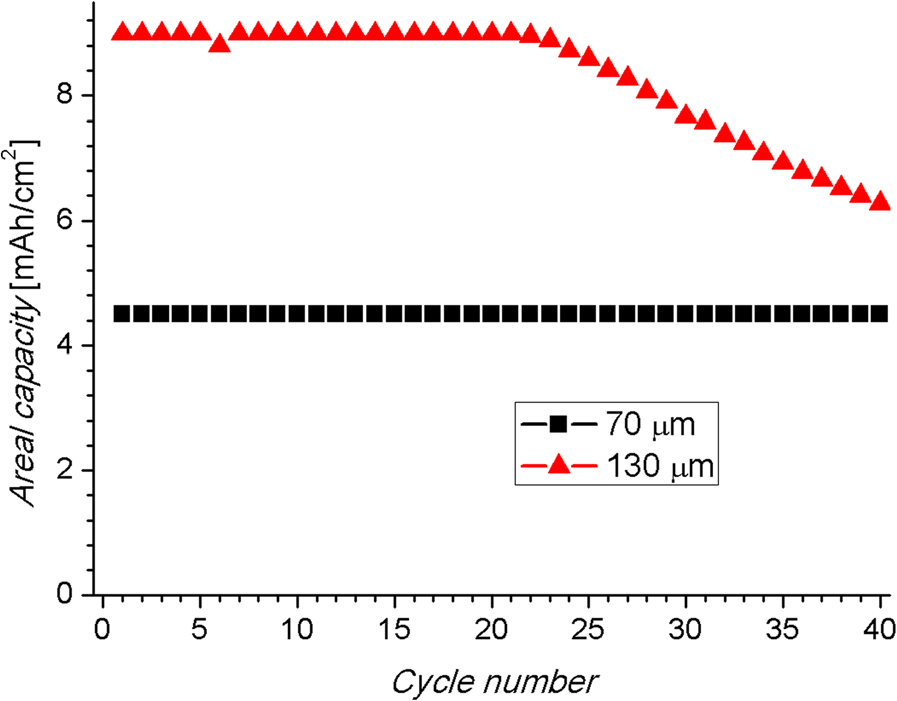
**Curve of areal capacity versus cycle number for anodes with wires of 70 and 130 μm.** The capacity of the anode with longer wires is two times the one with the shorter ones and is stable over 22 cycles. The first four cycles were performed at a cycling rate of C/10 and the rest at C/2.

The capacity of the anodes scales with the length of the wires. Figure 5 shows the lithiation capacity of anodes with wires of 70 and 130 μm over 40 cycles, cycling at a C rate of C/10 (the charging current is calculated so that the total capacity is reached in 10 h) for 4 cycles, and of C/2 afterwards, in galvanostatic/potentiostatic mode (see Methods section). To the side of the current collector, 10 μm of the anodes are embedded in Cu; this portion is not lithiated, since volume expansion is not allowed [11]. In this way, the active portion of the wires is of 60 and 120 μm, respectively. As expected, it can be observed in Figure 5 that the areal capacity (capacity per unit of area) of the anode with wires of 130 μm is around double the one of the anode with wires of 70 μm, before capacity fading. The areal capacity is directly proportional to the length of the wires.

### Performance limitations after scaling

**Figure 6:**
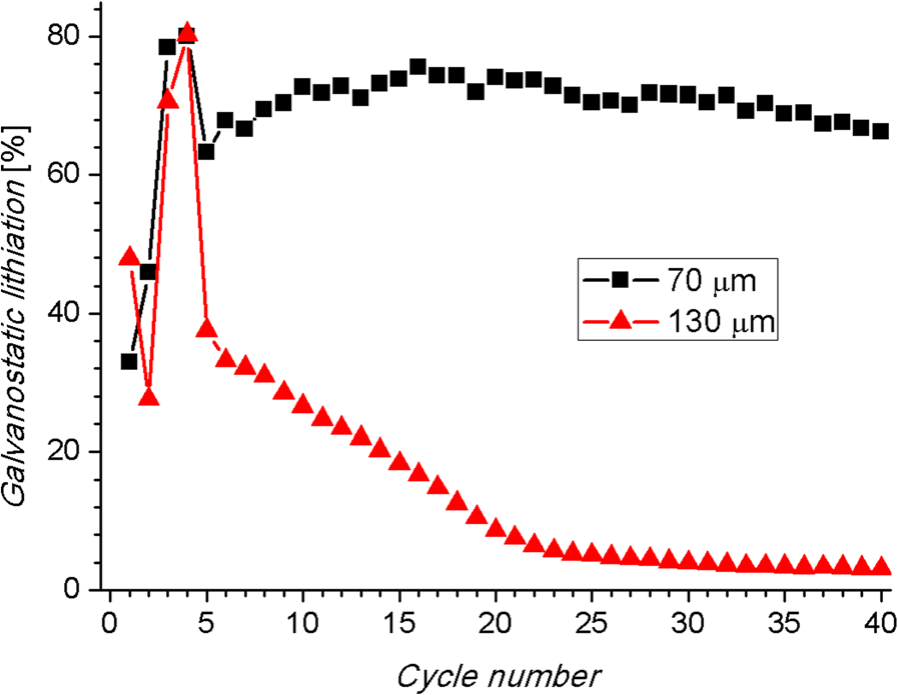
**Curves of the percentage of the lithiation capacity obtained galvanostatically.** The first four cycles were performed at a cycling rate of C/10 and the rest at C/2.

The increase of capacity after up-scaling has, however, a cost in the cyclability. The capacity of the longer wires fades monotonically after 22 cycles, as can be observed in Figure 5. The decrease of the capacity occurs most probably due to an increment in the series resistance. When the series resistance increases, the voltage limits defined for the cycling tests are reached faster. The increment in resistance can be produced by the diffusion limitation of the Li ions through the electrolyte among the wires (at high cycling rates) or by a continuous amorphization of Si upon cycling. The second effect is also known to occur in the shorter wires [10]. Nevertheless, in the case of longer wires, the increment in resistance at high cycling rates due to diffusion constraints is more significant.The previous statements can be corroborated when observing the percentage of the capacity obtained galvanostatically during the galvanostatic/potentiostatic cycling. As can be observed in Figure 6, during the first four cycles, when the cycling rate is C/10, the lithiation capacity obtained galvanostatically (galvanostatic lithiation) is similar in anodes with wires of 70 and 130 μm. The current density of C/10 is moderate, giving enough time to the Li ions to diffuse; thus, the most of the lithium storage (80%) is obtained galvanostatically. On the other hand, when the cycling rate is C/2, the Li diffusion is in its limit for the longer wires. With the diffusion limitation, the Li ions may be incorporated mainly at the wire tips, making the mean path for electrons and Li ions longer and, consequently, the mean electric resistance higher. As discussed before, when the resistance increases, the voltage limits are reached sooner, and the galvanostatic mode stops also sooner. That is why the percentage of charge is much lower for longer wires after cycle 5. Additionally, the capacity decreases continuously because in every delithiation cycle, some charge remains in the wires, and there is always less space for lithiation every cycle.

The amount of Li used for the formation of the solid electrolyte interface (SEI), normalized to the weight of Si, also scales when scaling the size of the wires. The sum of the irreversible Li losses (difference between the lithiation and delithiation capacities) during the first four cycles amounts to 1,606 mAh/g for the short wires and 3,087 mAh/g for the long wires (1.92 times the value for short wires). The SEI forms mainly during these first cycles, being the losses minimal afterwards. Considering the active portion of the wires with lengths 70 and 130 μm, the scaling factor is 2, value very close to the value 1.92 of the proportion of SEI. Thus, one may say that the SEI scales with the length, but tests with other wire lengths are necessary to confirm the theory. For the moment, the reason of this scaling is not clear. The SEI is an important structural component of the anode, which may be a decisive factor for the mechanical stability of the anode. Nevertheless, the amount of Li necessary to form it has to be carefully considered when scaling up the length of the wires; one needs an excess of Li of the order as the scaling factor.

## Conclusions

Producing Si microwire anodes out of macroporous Si is a fully scalable process. Mainly, the current for the electrochemical processes has to be scaled according to the desired area of the anodes. Having longer wires enables the storage of larger amount of charge per area (areal capacity), while larger anode areas represent larger amounts of active material and thus higher total capacities.

Scaling up the capacity pays, however, with a demerit in the performance of the anodes. Due to diffusion limitation of Li when scaling up the length of the wires, the capacity fades monotonically when cycling at high rates. On the other hand, the amount of Li necessary for the formation of the solid electrolyte interface scales up with the scaling factor.

## Competing interests

The authors declare that they have no competing interests.

## Authors' contributions

EQG prepared the samples for the study, made the battery tests, made the analysis of the results, and drafted the manuscript. JC contributed in the optimization of the fabrication of the battery anodes and helped in the analysis of the results and in the writing of the manuscript. HF participated in the coordination of the project and contributed in the analysis of the results and in the writing of the manuscript. All authors read and approved the final manuscript.

## Authors' information

EQG is a professor for materials science at the University of Puebla. He led the project for the development of high capacity Si wire anodes for Li ion batteries at the University of Kiel (‘general materials science’ group) until 2013. He is also a specialist in the synthesis and characterization of photoactive materials and microstructured electrodes for Li ion batteries. JC is a senior scientist in materials science. Since 1993, he coordinates the academic and scientific activities of the ‘general materials science’ group of the Institute for Materials Science of the University of Kiel. He is an expert in electrochemical pore etching in semiconductors, FFT impedance spectroscopy, and general characterization of solar cells. HF is a professor for materials science at the University of Kiel. He is the leader of the ‘general materials science’ group of the Institute for Materials Science. He is one of the co-finders of the electrochemical etching process of pores in n-type Si in 1990. His expertise includes silicides, electrochemical processes with semiconductors, and solar cells.
